# Predictive Value of Serum Uric Acid to HDL Cholesterol Ratio for Incident Ischemic Heart Disease in Non-Diabetic Koreans

**DOI:** 10.3390/biomedicines10061422

**Published:** 2022-06-15

**Authors:** Byoungjin Park, Dong-Hyuk Jung, Yong-Jae Lee

**Affiliations:** 1 Department of Family Medicine, Yongin Severance Hospital, Yongin-si 16995, Korea; bjpark96@yuhs.ac (B.P.); balsan2@yuhs.ac (D.-H.J.); 2 Department of Family Medicine, Gangnam Severance Hospital, Seoul 06273, Korea

**Keywords:** uric acid to HDL cholesterol ratio, cohort study, ischemic heart disease, Koreans

## Abstract

HDL cholesterol, besides its function in lipid metabolism, plays a role in suppressing blood oxidation reactions and protecting vascular endothelial cells. The uric acid/HDL cholesterol ratio (UHR) has recently attracted attention as a new biomarker for evaluating interactions between inflammatory and anti-inflammatory substances in the blood. This study aimed to investigate the longitudinal association between UHR and incident ischemic heart disease (IHD). Data from 16,455 participants without diabetes from the Health Risk Assessment Study (HERAS) and Korean Health Insurance Review and Assessment (HIRA) were assessed. Over 50 months after baseline enrolment, 321 (2.0%) participants developed IHD. The HRs of incident IHD were 0.85 (95% CI, 0.55–1.29), 1.42 (95% CI, 0.94–2.13), and 1.57 (95% CI, 1.01–2.45) in the second, third, and fourth UHR quartiles, respectively, after adjusting for potential confounding variables. In the subgroup analysis by sex-specific quartile, women tended to have higher HRs in the highest UHR quartile. We found that high UHR values were positively associated with incident IHD in Koreans without diabetes. An increased UHR may be a useful measure by which to assess cardiovascular risk in the preclinical stage.

## 1. Introduction

High-density lipoproteins (HDL) are noteworthy because they perform metabolic functions different from lipogenic lipoproteins [1]. Their importance as a biomarker for cardiovascular disease (CVD) needs to be understood in order to shed new light on various perspectives. HDL cholesterol removes excess cholesterol from peripheral tissues and transports it to the liver [2]. This process plays an essential role in redistributing cholesterol or removing cholesterol from the body through the gallbladder. 

In addition, HDL cholesterol plays a role in suppressing blood oxidation reactions and protecting vascular endothelial cells [3]. HDL cholesterol may interact directly or indirectly with other lipids and various substances in the blood. Low HDL cholesterol is a risk factor for atherosclerotic cardiovascular disease, but some concerns remain regarding the blood interaction between HDL cholesterol and oxidative stress-related substances. However, in a large-scale mendelian randomization study, increased HDL cholesterol was not associated with a beneficial effect on myocardial infarction risk, raising the question of HDL cholesterol as a biomarker alone for CVDs [4].

Uric acid is the end product of endogenous or dietary purine metabolism by xanthine oxidase [5]. Accumulated data show that increased uric acid can predict CVD morbidity and mortality even within the normal range [6,7,8]. Hyperuricemia is well known to increase gout development, but asymptomatic uric acid increases are also frequently found in insulin resistance, high blood pressure, and decreased renal function [9,10,11]. The activation of xanthine oxidase and the subsequent increase in uric acid production may be attributed to vascular endothelial dysfunction, increased advanced glycation end products formation, and the activation of the renin–angiotensin system (RAS), while inducing oxidative stress and chronic low-grade inflammation [12]. Nevertheless, controversy is ongoing between the cause and sequence between uric acid levels and CVD risks, and many studies show different effects between men and women [13,14].

Thus, we hypothesized that there is a continuous interaction between inflammatory and anti-inflammatory substances in the blood, and the two representative biomarkers are uric acid and HDL cholesterol, respectively. The uric acid/HDL cholesterol ratio (UHR) has recently attracted attention as a new biomarker for evaluating these interactions. Some studies have reported the relationship between UHR and diabetes and nonalcoholic fatty liver disease (NAFLD) [15,16], but few studies have reported the longitudinal association with CVD in the general population without diabetes. Thus, we aimed to examine the association between UHR and incident ischemic heart disease (IHD) in a large-scale, non-diabetic Korean population.

## 2. Materials and Methods

### 2.1. Participants and Data Collection

This study was derived from a Korean population-based cohort, the Health Risk Assessment Study (HERAS), and the Health Insurance Review and Assessment Service (HIRA) dataset. The aims and information of the HERAS–HIRA datasets were described in detail in previous studies. The cohort in urban areas of South Korea was enrolled to explore surrogate indicators for IHD through the collection of metabolic parameters and health-related behaviors. Initially, 20,530 adults were included in the baseline survey from November 2006 to June 2010. Subjects meeting any of the following criteria were excluded: previous diagnosis with IHD, ischemic stroke, or diabetes mellitus (including individuals with a fasting plasma glucose level ≥126 mg/dL); under 30 years of age; missing data; currently using aspirin or lipid-lowering medication; C-reactive protein ≥10 mg/L. Diabetes has been defined as a coronary heart disease equivalent according to the National Cholesterol Education Program (NCEP); individuals with diabetes often have asymptomatic angina, so they were excluded from the subjects of this study [17,18].

**Figure 1:**
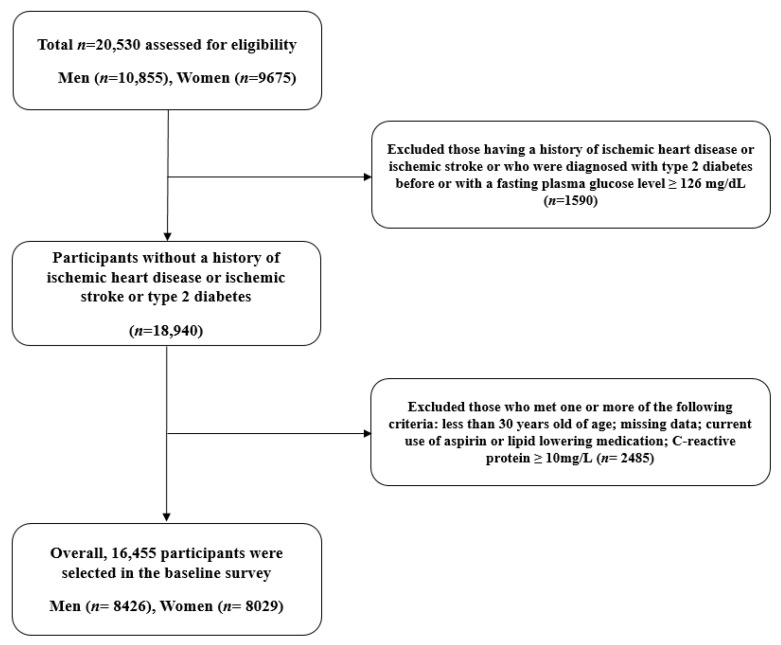
Flow chart of the study population selection.

After applying exclusion criteria, 16,455 individuals (8425 men and 8029 women) were included in the final analysis (Figure 1). The materials and methodology of the HERAS-HIRA datasets were described in detail in previous studies [19,20]. UHR (%) was calculated as uric acid (mg/dL) divided by HDL cholesterol (mg/dL). We divided the entire population according to the UHR quartiles as follows: Q1 ≤ 6.8 (≤25th percentile); Q2: ~9.3 (26 to 50th percentile); Q3: ~12.6 (51 to 75th percentile); and Q4 ≥ 12.6 (≥76th percentile). For the subgroup analysis, UHR values were additionally categorized into quartiles by men and women in the same way. The study protocol was approved by the institutional review board of the Yonsei University College of Medicine. Participant data were provided anonymously after the signing of an informed consent form.

### 2.2. Outcomes and Analysis

The outcomes were ischemic heart disease: angina pectoris (ICD-10 code I20) or acute myocardial infarction (ICD-10 code I21), which were assessed over the 50 months since the initial enrollment by linking each unique 13-digit identification number to the HIRA database. Demographic and biochemical characteristics were compared among the UHR quartiles using Pearson’s chi-squared test and analysis of variance for categorical and continuous variables, respectively. For C-reactive protein that does not exhibit a normal distribution, log transformation and analysis were performed. Age- and sex-adjusted survival curves were used to estimate the cumulative incidence of IHD for each group. After adjusting for potential confounding variables, we utilized the Cox proportional hazards regression model to assess the hazard ratios (HRs) and 95% confidence intervals (CIs) for incident IHD. We also calculated HRs and CIs for incident IHD according to sex-based UHR quartiles in the same way. All statistical analyses were performed using SAS software (version 9.4; SAS Institute Inc., Cary, NC, USA). Statistical significance was set at *p* < 0.05. 

## 3. Results

Table 1 shows the demographic and biochemical characteristics of the HERAS–HIRA cohorts based on UHR quartiles (*n* = 16,455; 8426 men and 8029 women). The mean age, body mass index, and UHR were 46.1 ± 9.5 years, 23.4 ± 3.0 kg/m^2^, and 10.1 ± 4.2%, respectively. The mean values of mean arterial pressure, fasting plasma glucose, total cholesterol, triglyceride, and Log C-reactive protein levels were highest in the group with the highest UHR quartile. This group also exhibited the highest proportions of alcohol drinkers and current smokers while showing the lowest proportion of individuals involved in regular exercise. According to the UHR quartiles, the prevalence of impaired fasting glucose, metabolic syndrome, and hypertension were 18.0%, 12.1%, and 20.3%, respectively. Finally, the higher the UHR quartile, the more significant the male proportion was. 

During the follow-up period, 321 (2.0%, 321/16,455) individuals developed IHD. Multivariate Cox regression analysis models were used to evaluate the relative contribution of the UHR quartile to the development of IHD (Table 2). Compared with the first UHR reference quartile, the HRs of incident IHD were 0.84 (95% CI, 0.55–1.29), 1.43 (95% CI, 0.95–2.15), and 1.57 (95% CI, 1.01–2.44) in the second, third, and fourth UHR quartiles, respectively, after adjusting for age, sex, body mass index, smoking status, alcohol intake, and physical activity. Similarly, these longitudinal associations remained after additionally adjusting for mean arterial blood pressure, fasting plasma glucose, and Log C-reactive protein level. 

**Figure 2:**
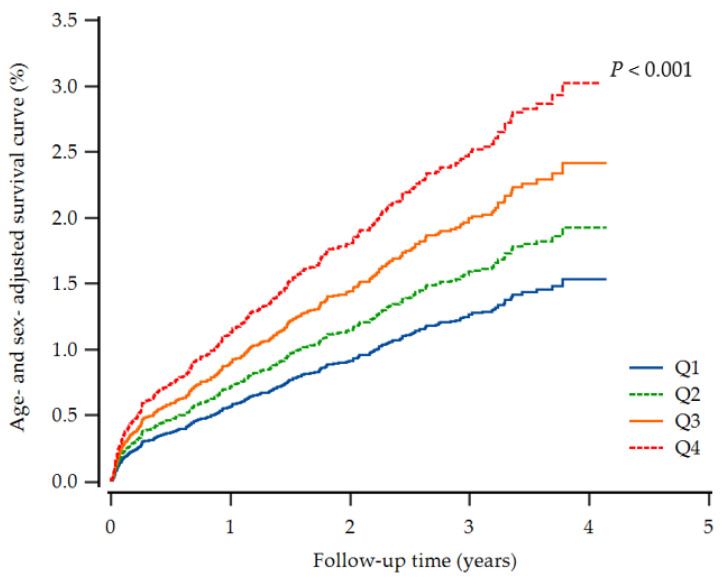
Cox regression survival curve for new-onset ischemic heart disease.

Furthermore, the highest UHR group showed a higher cumulative incidence of IHD up to 50 months after adjusting for age and sex (*p* < 0.001) (Figure 2). In the subgroup analysis by sex-specific quartile, women tended to have higher HRs in the highest UHR quartile (Table 3). 

## 4. Discussion

This large-scale cohort study on non-diabetic Koreans found that high UHR values were positively associated with incident IHD, which remained after adjustment for potential confounding factors, including health behaviors and hypertension medication. In the sex-specific analysis, women tended to have a higher risk for IHD in the highest UHR quartile.

Previous studies have reported that UHR is useful for predicting metabolic syndrome. Kocak et al. reported that serum UHR is a valuable predictor of metabolic syndrome in Turkish people with diabetes [21], while Yazdi et al. reported that UHR could screen or diagnose metabolic syndrome risks in Iranians without diabetes [22]. Aktas et al. have suggested that UHR can be used for diabetes control evaluations in men with diabetes [15]. In addition, Kosekli et al. reported the relationship between UHR and nonalcoholic liver disease in a single institution study [23], and a cross-sectional study among lean Chinese also reported the relationship between UHR and nonalcoholic liver disease [16]. Moreover, a recent epidemiological study has also shown that UHR can increase the inflammatory burden [24]. Our study was conducted longitudinally and identified associations between UHR and new-onset IHD among non-diabetic individuals, which may result from the accumulation of metabolic or inflammatory changes.

Uric acid is the breakdown end-product of purine metabolism, controlled by an enzyme known as xanthine oxidase. Uric acid is well known to cause gout arthritis, but it is commonly increased in patients with metabolic syndrome or diabetes mellitus [25]. Some animal studies have reported that hyperuricemia can reduce insulin sensitivity by increasing reactive oxidative stress and inflammatory cytokines such as TNF-alpha [26,27]. In addition, it can be accompanied by a reduction in nitrogen oxide and RAS activation, which can have adverse effects on vascular health [28]. Some studies have reported that taking uric acid lowering agents, such as a xanthine oxidase inhibitor and uricosuric agent, helped improve insulin resistance and high blood pressure [29,30]. However, the number of participants is small, and there is a limitation in the short-term follow-up study. The lack of evidence through a well-designed randomized controlled study raises the need for an understanding of interactions with anti-inflammatory substances in the blood. 

HDL cholesterol plays a crucial role in promoting cholesterol efflux in arterial wall cells, reducing the accumulation of foam cells, and suppressing the oxidation of LDL cholesterol to alleviate atherogenicity [31]. HDL cholesterol also has a variety of antioxidants and anti-inflammatory agents which prevent arteriosclerosis in circulation. ApoA1, ApoA2, and paraoxonase of HDL are major antioxidant and anti-inflammatory components [32]. HDL reduces the synthesis of endothelium adhesion proteins induced by C-reactive protein or proinflammatory cytokines [33,34]. In this study, as the UHR quartiles increased, the prevalence of impaired fasting glucose and metabolic syndrome gradually increased, and the inflammatory index and C-reactive protein tended to increase. This suggests the possibility that UHR values can predict IHD by reflecting insulin sensitivity and inflammatory and anti-inflammatory conditions.

According to the NCEP definition, the HDL cholesterol criteria for metabolic syndrome differs between men and women [35]. Women also tend to have lower uric acid levels than men due to estrogen’s uricosuric effect [14]. Previous studies have shown that increased uric acid in women has been associated with higher cardiometabolic morbidity [36,37,38]. The plausible mechanism suggests insulin resistance burden in different sexes according to uric acid levels [14,39]. We evaluated the interaction between uric acid and HDL cholesterol; IHD incidence increased with the higher UHR in an age- and sex-adjusted survival curve, and when sex-specific UHR was applied, the new-onset IHD risk was higher in women in the highest quartile.

The primary strength of our study is that this was a large-scale longitudinal study linked to HIRA data based on Korea’s universal coverage system, thereby reducing the likelihood of missing data. However, this study had some limitations that should also be acknowledged. First, some confounding variables, including dietary factors and traditional insulin resistance markers, may not have been initially evaluated. Second, since hemoglobin A1c evaluation and oral glucose tolerance tests were not performed in the baseline survey, some individuals with diabetes may have been included in the final analysis.

## 5. Conclusions

UHR values positively and significantly predicted future IHD in a large-scale cohort study on Koreans without diabetes, reflecting insulin resistance combined with chronic inflammation. In addition, women tended to have more risk for IHD at high UHR levels. Accordingly, increased UHR may be a useful additional measure by which to assess cardiovascular risk for non-diabetic adults in the preclinical stage. 

## Figures and Tables

**Table 1:** Baseline characteristics of the study population.

	Q1 (*n* = 4113)	Q2 (*n* = 4114)	Q3 (*n* = 4114)	Q4 (*n* = 4114)	*p* Value ^1^	Post Hoc ^2^
UHR (%)	≤6.8	~9.3	~12.6	≥12.6		
Age (years)	45.2 ± 9.1	46.6 ± 9.7	46.8 ± 9.6	45.7 ± 9.5	<0.001	a,b,e,f
Male sex (%)	9.7	32.8	70.2	92.2	<0.001	-
Body mass index (kg/m^2^)	21.6 ± 2.6	22.7 ± 2.7	23.9 ± 2.7	25.2 ± 2.7	<0.001	a,b,c,d,e,f
Systolic blood pressure (mmHg)	116.3 ± 15.0	120.0 ± 15.5	124.3 ± 14.7	127.2 ± 14.5	<0.001	a,b,c,d,e,f
Diastolic blood pressure (mmHg)	72.2 ± 9.6	74.9 ± 10.0	77.9 ± 9.6	79.9 ± 9.4	<0.001	a,b,c,d,e,f
Fasting plasma glucose (mg/dL)	88.3 ± 8.7	90.2 ± 9.2	92.9 ± 10.0	94.3 ± 10.0	<0.001	a,b,c,d,e,f
Uric acid (mg/dL)	3.6 ± 0.7	4.4 ± 0.7	5.4 ± 0.8	6.5 ± 1.0	<0.001	a,b,c,d,e,f
Total cholesterol (mg/dL)	187.9 ± 31.7	188.3 ± 33.4	192.2 ± 33.8	192.6 ± 34.0	<0.001	b,c,d,e
Triglyceride (mg/dL)	81.6 ± 35.7	102.2 ± 50.1	131.4 ± 72.0	181.6 ± 119.4	<0.001	a,b,c,d,e,f
HDL cholesterol (mg/dL)	66.3 ± 11.3	55.7 ± 8.9	49.5 ± 7.6	41.4 ± 6.4	<0.001	a,b,c,d,e,f
Log C-reactive protein (mg/L)	−1.0 ± 1.0	−0.7 ± 1.0	−0.4 ± 1.0	0.0 ± 0.9	<0.001	a,b,c,d,e,f
Current smoker (%)	8.0	16.2	31.2	42.6	<0.001	-
Alcohol drinking ^3^ (%)	30.3	36.2	50.0	56.4	<0.001	-
Regular exercise ^4^ (%)	31.3	32.0	32.1	28.3	<0.001	-
Impaired fasting glucose (%)	9.2	14.3	22.0	26.5	<0.001	-
Metabolic syndrome (%)	2.3	7.7	13.4	25.1	<0.001	-
Hypertension (%)	11.1	17.4	22.9	29.9	<0.001	-

^1^ *p*-values were calculated using one-way ANOVA or Pearson’s chi-squared test. ^2^ Post hoc analysis with the Bonferroni method: a, Q1 versus Q2; b, Q1 versus Q3; c, Q1 versus Q4; d, Q2 versus Q3; e, Q2 versus Q4; f, Q3 versus Q4. ^3^ Alcohol consumption ≥ 140 g of ethanol/week. ^4^ Moderate intensity physical exercise ≥ three times/week.

**Table 2:** Hazard ratios (95% confidence intervals) for incident ischemic heart diseases.

		Q1	Q2	Q3	Q4	*p* for Trend
New cases of ischemic heart disease, *n*		56	52	98	115	
Mean follow-up, years		2.4 ± 1.1	2.4 ± 1.1	2.4 ± 1.1	2.4 ± 1.1	
Pearson–years of follow-up		9778	9713	9716	9825	
Incidence rate/1000 person–years		5.7	5.4	10.1	11.7	
Model 1	HR (95% CI)	1.00 (reference)	0.88 (0.58–1.34)	1.55 (1.04–2.31)	1.78 (1.17–2.70)	0.001
	*p* value	-	0.548	0.031	0.006	
Model 2	HR (95% CI)	1.00 (reference)	0.84 (0.55–1.29)	1.43 (0.95–2.15)	1.57 (1.01–2.44)	0.009
	*p* value	-	0.425	0.089	0.044	
Model 3	HR (95% CI)	1.00 (reference)	0.85 (0.55–1.29)	1.42 (0.94–2.13)	1.57 (1.01–2.45)	0.011
	*p* value	-	0.436	0.097	0.045	

Model 1: adjusted for age and sex. Model 2: adjusted for age, sex, body mass index, smoking status, alcohol intake, and physical activity. Model 3: adjusted for age, sex, body mass index, smoking status, alcohol intake, physical activity, mean arterial blood pressure, fasting plasma glucose, and Log C-reactive protein level.

**Table 3:** Hazard ratios (95% confidence intervals) for incident ischemic heart diseases according to sex-specific UHR.

Men		Q1 (≤9.8)	Q2 (~12.2)	Q3 (~14.9)	Q4 (≥14.9)	*p* for Trend
Model 1	HR (95% CI)	1.00 (reference)	1.44 (0.94–2.22)	1.73 (1.14–2.63)	1.71 (1.12–2.61)	0.045
	*p* value	-	0.096	0.009	0.013	
Model 2	HR (95% CI)	1.00 (reference)	1.38 (0.90–2.14)	1.61 (1.05–2.45)	1.54 (0.98–2.40)	0.156
	*p* value	-	0.143	0.029	0.059	
Model 2	HR (95% CI)	1.00 (reference)	1.38 (0.89–2.14)	1.61 (1.05–2.47)	1.55 (0.99–2.43)	0.155
	*p* value	-	0.145	0.029	0.056	
**Women**		**Q1** **(≤5.7)**	**Q2** **(~7.0)**	**Q3** **(~8.7)**	**Q4** **(≥8.7)**	***p* for Trend**
Model 1	HR (95% CI)	1.00 (reference)	1.53 (0.78–3.01)	1.38 (0.70–2.72)	2.07 (1.12–3.85)	0.113
	*p* value	-	0.216	0.350	0.021	
Model 2	HR (95% CI)	1.00 (reference)	1.53 (0.78–3.00)	1.38 (0.70–2.73)	2.02 (1.06–3.84)	0.167
	*p* value	-	0.221	0.355	0.032	
Model 3	HR (95% CI)	1.00 (reference)	1.52 (0.77–2.99)	1.39 (0.70–2.76)	2.01 (1.06–3.84)	0.180
	*p* value	-	0.225	0.342	0.033	

Model 1: adjusted for age. Model 2: adjusted for age, body mass index, smoking status, alcohol intake, and physical activity. Model 3: adjusted for age, body mass index, smoking status, alcohol intake, physical activity, mean arterial blood pressure, fasting plasma glucose, and Log C-reactive protein level.

## Data Availability

The data underlying this article will be shared upon reasonable request from the corresponding author.
